# Quitting Shisha Smoking Among Young Adults in Germany: Insights From an Online Survey on Sociodemographic, Perceptual and Behavioural Economic Factors Associated With Quit Attempts

**DOI:** 10.1177/1179173X261454173

**Published:** 2026-08-07

**Authors:** Cynthia Fedler, Daniel Kotz, Markus Vomhof, Stephanie Klosterhalfen

**Affiliations:** 1 Institute of General Practice (ifam), Centre for Health and Society (chs), Addiction Research and Clinical Epidemiology Unit, Medical Faculty and University Hospital Düsseldorf, 9170 Heinrich-Heine-University Düsseldorf, Germany; 2 Department of Behavioural Science and Health, 39064 University College London, London, UK; 3 Institute for Health Services Research and Health Economics, Centre for Health and Society, Medical Faculty and University Hospital Düsseldorf, Heinrich-Heine-University Düsseldorf, Düsseldorf, Germany; 4 Institute for Health Services Research and Health Economics, German Diabetes Center, Leibniz Center for Diabetes Research at Heinrich-Heine-University Düsseldorf, Düsseldorf, Germany

**Keywords:** shisha, waterpipe, quit attempt, risk preferences, time preferences

## Abstract

**Introduction:**

Shisha smoking is common among young adults despite well-documented health risks. Evidence on factors linked to quit attempts among shisha smokers is limited. We examined sociodemographic, consumption-related, harm-perception, and behavioural-economic correlates of quit attempts in Germany.

**Methods:**

We analysed baseline data from the SHISHA study (01/2024–12/2025), a pilot online survey of adults aged 18–40 years in Germany. Participants were recruited via social media, schools, and flyers. Associations with quit attempts were examined using univariable logistic regressions.

**Results:**

Of 1,134 respondents, 570 identified themselves as current shisha smokers, and 504 provided valid data on quit attempts. The median age was 24 years; 68.3% were male and 30.2% female. Overall, 14.6% (95% CI = 11.9–17.8) of current shisha smokers reported having ever attempted to quit. Perceived dependence was the only variable significantly associated with quit attempts: those rating it unlikely to be or become addicted had higher odds of having attempted to quit than those rating it likely (OR = 2.05, 95% CI: 1.26–3.32). No significant associations were observed for sociodemographic characteristics, consumption patterns, perceived health risk, or behavioural-economic measures; descriptively, quit attempters showed more risk-averse choices.

**Discussion:**

In Germany, people who smoke shisha are typically young adults and have never attempted to quit. The observed association between low perceived dependence and the absence of quit attempts leaves open whether perceived dependence drives cessation behaviour or whether previous quit attempts shape individuals’ perception of their own dependence.

## 1. Introduction

Shisha tobacco smoking remains widespread, particularly among young adults, despite mounting evidence of its health risks. Recent data from Germany indicate that approximately 6% of individuals aged 14 to 24 years currently smoke shisha.^1^ Understanding how current shisha smokers perceive risks and perceived dependence is essential, as these beliefs may influence both, motivation and behaviour, related to quitting.^2^ Within a broader and increasingly diverse landscape of tobacco and nicotine products, these perceptions may be shaped differently compared to conventional cigarette smoking. Many shisha smokers consider their consumption relatively harmless^3^ and express high confidence in their ability to quit at any time. This combination may contribute to the low prevalence of quit attempts in this population.^4^ While cigarette smoking has declined across many high-income countries, shisha use remains prevalent among young adults in Europe,^5,6^ yet cessation interventions remain limited in Germany.

Scientific evidence highlights that shisha smoking is associated with substantial health risks including impaired lung function, elevated heart rate, increased risk of cardiovascular disease, cancer, and chronic respiratory illness.^7,8^ Compared to cigarette smoking, a single shisha session involves approximately 74 litres of smoke inhalation, vs. 0.6 litres per cigarette, and leads to significantly higher carbon monoxide exposure.^9^ Shisha tobacco usually contains nicotine and is associated with a high risk of dependence.^10,11^ The combination of smooth, cooled smoke and sweet flavours contributes to the common perception that shisha smoking is less harmful than cigarette use.^12^

The current evidence base on determinants of quit attempts among shisha smokers remains limited. There is no clear evidence that socio-demographic factors are associated with quit attempts in this group. Preliminary findings for shisha use indicate that certain consumption-related factors might be associated with a higher likelihood of a quit attempt. Shisha smokers with shorter smoking histories and lower consumption frequency are more likely to express intentions to quit.^13,14^ Furthermore, existing studies suggest that exclusive shisha smokers are more likely to attempt quitting compared to poly tobacco users, who tend to exhibit lower cessation rates and higher nicotine dependence.^15,16^ In Germany, most people who smoke shisha do not use shisha exclusively — dual and multiple product use are common.^17^ In terms of harm perception, smokers who perceived themselves as more dependent tend to report lower confidence in their ability to quit.^13^ Moreover, shisha smokers who perceive greater health risk from their shisha smoking are more motivated to quit.^18^ Despite this self-reported motivation, actual quit attempts appear to be relatively rare among people who smoke shisha. Factors such as low perceived harm, social acceptance, and pleasurable characteristics such as sweet flavours may contribute to this discrepancy. The social nature of shisha use - typically shared in cafés or private gatherings – and its embedding in social and cultural practices may further reinforce continued use and reduce motivation to quit. However, existing research has not yet systematically explored the full range of individual and contextual factors that might predict quit attempts in this population.

Beyond these established factors, individual decision-making styles may also influence smoking behaviour. This may be especially relevant for shisha use, which is often social, ritualised, and perceived as a leisure activity rather than a form of nicotine dependence. Such characteristics might shape decision-making processes differently from cigarette smoking. Two behavioural health economic concepts are particularly relevant in this context**:** risk preferences, which describe attitudes towards taking risks (i.e. choosing between a riskier but smaller payment vs. less risky but higher payment) and time preferences, which reflects how immediate smaller rewards are weighted against delayed larger ones. These economic preferences have been shown to influence a range of health-related behaviours, including smoking behaviour.^19-21^ Research on cigarette smoking suggests that behavioural health economic time and risk preferences may help explain why some smokers attempt to quit while others do not. Several studies have shown that individuals who are more present-oriented or more willing to take risks are significantly more likely to smoke and less likely to quit.^22,23^ Smokers consistently display steeper delay discounting than non-smokers, indicating a stronger preference for immediate rewards.^24,25^ Moreover, longitudinal findings suggest that high discounting can precede smoking initiation and predict continued use.^26^ Delay discounting has also been linked to lower engagement with anti-smoking campaigns, reduced risk perception, and fewer quit attempts.^27,28^ Whether these behavioural tendencies similarly shape cessation behaviour among people who smoke shisha remains an open question, particularly given potential differences in motivations, social context, and risk perception.

To date, no study has jointly examined sociodemographic, consumption-related, harm-perception and behavioural-economic determinants of quit attempts among shisha smokers.

Building on this framework, the present study examines factors associated with quit attempts among shisha smokers in Germany. Specifically, we investigate whether these factors are associated with cessation behaviour. By integrating these domains, the study provides novel evidence on behavioural determinants of shisha smoking cessation.

## 2. Methods

### 2.1. Study Design and Participants

This study is based on data from the SHISHA study (**S**ocial **H**abits and **I**nsights in Shi**sha** use), a pilot longitudinal online panel survey conducted in Germany (January 2024 - December 2025). The primary objectives of this pilot were to test recruitment strategies, to refine an online questionnaire, and to gather first insights into shisha smoking patterns among young adults. The SHISHA study follows a repeated-measures design, with a baseline survey open to all participants. At baseline, current shisha smokers could leave contact details for two shorter follow-up surveys conducted at three-month intervals (3 months and 6 months after baseline). For follow-up waves, eligibility was restricted to participants who reported current waterpipe use at baseline. Inclusion criteria at baseline were age ≥14 years, provision of informed consent, and sufficient German language proficiency to complete the questionnaire. Informed consent was obtained electronically within the online questionnaire by requiring participants to actively confirm their agreement before proceeding. For participants aged under 18 years, written informed consent from both the adolescents and their parents or legal guardians was additionally required prior to participation. The study received ethical approval from the Ethics Committee of the Medical Faculty, Heinrich Heine University Düsseldorf (2023-2396) and was registered with the German Clinical Trials Register (DRKS00033530); all participants gave informed consent. The study design and procedures are described in detail in the published study protocol, available on the Open Science Framework (https://osf.io/gvq37/files/wtk9j).

Before the survey launched, the questionnaire was pre-tested with individuals from the target population, and minor adjustments were implemented based on their feedback. Survey data were collected using structured online questionnaires at baseline and follow-up assessments.

Participants were recruited for the SHISHA study through a combination of online and offline strategies, aiming to reach a diverse sample of current shisha smokers aged 14 to 40 years, though older individuals could also participate. For the present analysis, only participants aged 18 years and older were included. Recruitment channels included social media platforms, flyers with QR codes distributed in shisha bars, a population survey,^29^ and collaboration with schools. To support retention, participants were entered into a lottery for 100 25€ digital vouchers, with half distributed before and half after follow-up surveys.

### 2.2 Measurements

Current shisha smoking was measured by asking: “Do you smoke shisha (also known as a waterpipe)?”, with response options: (a) “Yes, still today” [defines current smoking], (b) “Yes, regularly, but I am not currently smoking”, (c) “Yes, I used to, but I am not currently smoking”, (d) “No, I have never smoked shisha”, and (e) “I prefer not to answer”.

Quit attempts were captured with the question: “Have you ever tried to quit smoking shisha?”, with response categories: (a) “Yes, I have tried at least once”, (b) “No, I have not tried yet”, and (c) “No, I have not thought about it yet”.

Sociodemographic variables included age (continuous), gender, monthly disposable income, educational qualification, and migration background. Gender was self-reported with the options female, male, diverse, and prefer not to say, and was recoded into male/female for analysis. Monthly disposable income was reported in six predefined income brackets and grouped into <€1,001, €1,001–2,000, and >€2,000. Educational qualification was reported using standard German schooling categories and summarised into low (≤9 years of schooling), medium (≥10 years), and high (≥12 years). Migration background was derived from parental country of birth (both born in Germany/only one/neither/prefer not to say) and dichotomised into yes/no, where “yes” indicated that at least one parent was born outside Germany.

Consumption-related factors covered consumption quantity (number of heads per month) and concurrent use of other nicotine- or tobacco-containing products (e.g. cigarettes, e-cigarettes, heated tobacco products, cigarillos, snus).

Health perception was measured with two items. While comprehensive approaches to assessing dependence in users of new tobacco and nicotine products have been proposed, including recent consensus recommendations,^30^ we used a brief measure of self-perceived dependence to reduce respondent burden in this pilot study.

(1) Self-perceived dependence was measured with the question “How likely do you think it is that you could become or have become addicted to shisha tobacco?”, with four response options ranging from “very unlikely” to “very likely” For regression analysis, we dichotomised responses into “unlikely” (very/rather unlikely) and “likely” (rather/very likely).
(2) Perceived health risks were assessed by asking: “Do you think smoking shisha harms your health?”, with four response options: “no”, “yes, it harms my health a little”, “yes, it harms my health somewhat”, “yes, it harms my health a lot”; responses were dichotomised into “not harmful” (response 1) and “harmful” (responses 2-4).

Risk preferences were measured by a multiple price list (MPL) framework similar to Holt and Laury (2002).^31^ To provide a representation as closely as possible to the realities of the respondents, choice tasks were represented by wheels of fortune (see Supplementary Figure 1). This visual format, developed by the study team, was an adaptation of commonly used pie charts to represent lottery choices in risk preference elicitation.^19,32^ In particular, participants saw ten pairs of choice tasks, each requiring a decision between two hypothetical wheels of fortune: one with a smaller difference in payoffs (€160 vs. €200) and one with a larger difference in payoffs (€10 vs. €385). The Payoffs were based on Holt and Laury’s values, multiplied by 100 to provide realistic amounts without decimals. The probability is captured by the share of 10 segments associated with the payoffs. The number of segments associated with the lower payoff increased stepwise from one to ten, while the corresponding number of higher-payoff segments decreased accordingly—on both wheels. Participants were instructed to indicate which wheel they would choose in each choice task. Based on their switching point, participants were categorised as risk-averse (switching point 1-5) or risk-seeking (switching point 6-10). No real incentives were used, but the framing included a light tone to enhance engagement. Note, that our MPL differs from that one of Holt and Laury which increased stepwise the probability for the higher payoffs. As a result, the number of choice tasks corresponding to risk-aversion is greater by one in our version. Individuals with incomplete risk preference data, with more than one switch point or choosing the lower certain payment in the last choice (internal validity test) were excluded from analysis.

Time preferences were assessed only among participants who agreed to complete a short set of additional questions at the end of the main survey. They were elicited in a MPL format.^33,34^ In our MPL, participants were asked to decide between receiving €300 immediately or a larger reward after a delay. The task included two sets of eight choices: in the first block, the delayed reward was available after one month, and in the second block after six months. Within each block, the value of the delayed reward increased progressively across items (from €307 to €356), allowing participants’ switching point to be identified—that is, the moment they preferred the delayed over the immediate reward. Based on their switching point, participants were categorised into three groups: future-oriented (those who chose the delayed reward in each choice), variable discounting (those who switched once from the immediate to the delayed option), and present-oriented (those who chose the immediate reward in each choice). The task was hypothetical and presented in a tabular format. Individuals with incomplete time preference data or with more than one switch point were excluded from analysis.

### 2.3. Data Analysis

We used descriptive statistics to summarise the prevalence of quit attempts, participant characteristics, health perceptions and behavioural variables. Percentages are reported with corresponding 95% confidence intervals (CI).

To analyse factors associated with quit attempts, we conducted a series of univariable logistic regression models, with quit attempt (yes vs. no) as the dependent variable. Independent variables included age (continuous), gender (male/female), income (low/medium/high), educational qualification (low/medium/high), migration background (yes/no), consumption quantity (continuous), concurrent use of other tobacco or nicotine products (yes/no), perceived dependence (unlikely/likely), perceived health risks (not harmful/harmful), risk preferences (risk-averse/seeking), and time preferences (present-/variable-/future-oriented). All analyses were based on complete cases and unweighted data and performed using IBM SPSS Statistics (version 29; IBM Corp.).

## 3. Results

At baseline, 1,134 individuals completed the online-survey conducted between August and November 2024. Among them, 50.3% (n=570) identified themselves as current shisha smokers. Of these, 88.4% (n=504) answered the question about quit attempts.

Baseline characteristics of the total sample and the subsample of current shisha smokers are shown in Table 1. The median age in both groups was 24 years, and the proportion with a migration background was similar (37.4% vs. 37.9%). Compared with the overall sample, current shisha smokers were more often male (83.2%, 95% CI = 79.8-86.1 vs. 68.3%, 95% CI = 69.3-74.5) and showed a different distribution of educational qualification, with fewer reporting a high level (59.8%, 95% CI = 55.7-63.7 vs. 72.0%, 95% CI = 69.3-74.6) and more a middle level (26.8%, 95% CI = 23.2-30.7 vs. 16.9%, 95% CI = 14.8-19.2). They were also somewhat more likely to report middle or high income compared with the overall sample.

**Table 1.** Baseline Sociodemographic Characteristics of Young Adults in the SHISHA Study

Characteristic % (n)	Total 100 (1,079)	95% CI	Current Shisha Smoker*ꟸ* 52.8 (570)	95% CI
**Years of age, median (25%, 75%), n=1134** ^**#**^	24 (20, 27)	​	24 (21, 29)	​
**Sex**				
Male	68.3 (723)	65.6-71.0	83.2 (474)	79.8-86.1
Female	30.2 (343)	27.5-33.0	11.9 (68)	9.5-14.8
**Educational qualification** ^*✜*^				
Low	6.3 (71)	5.0-8.0	9.3 (53)	7.1-12.0
Middle	16.9 (192)	14.8-19.2	26.8 (153)	23.2-30.7
High	72.0 (816)	69.3-74.5	59.8 (341)	55.7-63.7
**Income** ^*’*^				
Low (<€1,001)	49.8 (565)	46.8-52.8	41.1 (234)	37.0-45.4
Middle (€1,001-2,000)	19.4 (220)	17.1-21.9	24.7 (141)	21.2-28.5
High (>€2,000)	19.7 (223)	17.3-22.2	24.2 (138)	20.7-28.0
**Migration background** ^*^				
Yes	37.4 (424)	34.6-40.3	37.9 (216)	33.9-42.0
No	55.3 (627)	52.3-58.2	54.9 (313)	50.7-59.0

Data are shown as percentages (absolute numbers). The total sample is represented as 100% within each column. Any discrepancies in the total column percentage are due to missing data for the respective variable.

^#^Higher valid n for age reflects early survey drop-out, as the age item was positioned at the beginning to screen participants for eligibility.

ꟸSubgroup of the total sample.

^✜^German equivalents to education qualification listed from lowest to highest: low=no qualification/junior high school equivalent, middle=secondary school equivalent, high=advanced technical college equivalent/high school equivalent.

^’^Net monthly household income: low=<€1,000, middle=€1,001-2,000), and high=>€2,000.

*Migration background: Yes= one or both parents born abroad.

Among current shisha smokers, the median consumption quantity was 12 sessions per month (IQR: 5–30). About 39% (95% CI = 11.9-17.8) reported concurrent use of at least one other tobacco or nicotine product. 14.6% (95% CI = 11.9-17.8) had ever made a quit attempt, while the majority had not tried or had not yet thought about quitting. Perceptions of dependence were mostly in the “rather unlikely” (31.1%, 95% CI = 27.3-35.1) or “very unlikely” (24.0%, 95% CI = 20.6-27.7) categories, whereas smaller proportions considered dependence “rather likely” (18.8%, 95% CI = 15.8-22.1) or “very likely” (11.8%, 95% CI = 9.4-14.7). More than three quarters acknowledged at least some health risks associated with shisha smoking (Table 2)**.**

**Table 2.** Smoking Behaviour and Behavioural-Economic Characteristics Among Current Shisha Smokers

Characteristic	Current Shisha Smoker 100 (570), % (n)	95% CI
**Shisha sessions per month** *ꟸ,* **median (25%, 75%)**	12 (5, 30)	​
**Age of initiation**		
≤15 years	24.6 (128)	21.1-28.4
16-18 years	58.0 (302)	53.7-62.1
≥19 years	17.5 (91)	14.4-21.0
**Concurrent use of additional nicotine products** ^*✜*^		
cYes	38.9 (222)	34.9-43.1
No	61.1 (348)	56.9-65.1
**Quit Attempts**		
Yes, I have tried at least once.	14.6 (83)	13.4-20.1
No, I have not tried yet.	33.3 (190)	33.4-42.1
No, I have not thought about it yet.	40.5 (231)	41.3-50.3
**Perceived Dependence**		
Very likely	11.8 (67)	10.6-16.6
Rather likely	18.8 (107)	17.8-25.0
Rather unlikely	31.1 (177)	31.0-39.2
Very unlikely	24.0 (137)	23.4-31.1
I don’t know	3.0 (17)	2.1-5.5
**Perceived Health Risks**		
No	10.0 (57)	8.8-14.5
Yes, it harms my health a little	27.0 (154)	26.7-34.7
Yes, it harms my health somewhat	35.4 (202)	35.8-44.3
Yes, it harms my health a lot	13.7 (78)	12.4-18.9
I don’t know	2.5 (14)	1.7-4.7
**Risk preferences** ^#^		
Risk averse	84.8 (302)	80.7-88.2
Risk seeking	15.2 (54)	11.8-19.3
**Time preferences 1 Month** ^*^		
Future-oriented	44.1 (138)	38.6-49.7
Variable discounting	45.0 (141)	39.4-50.6
Present-oriented	10.9 (34)	7.8-15.0
**Time preferences 6 Month** ^*^		
Future-oriented	22.0 (69)	17.4-26.6
Variable discounting	40.9 (128)	35.4-46.4
Present-oriented	37.1 (116)	31.7-42.5

ꟸ **Consumption quantity:** Number of shisha heads used per month.

^✜^ **Concurrent use**: Use of at least one additional product (cigarettes, e-cigarettes, or heated tobacco).

^#^**Risk preferences: **classified as risk averse (switching point 1-5) or risk seeking (switching point 6-10).

*Time preferences (1- and 6-month task): future-oriented= always chose delayed reward, variable discounting=switched once, present-oriented=always chose delayed reward.

**Figure 1.**
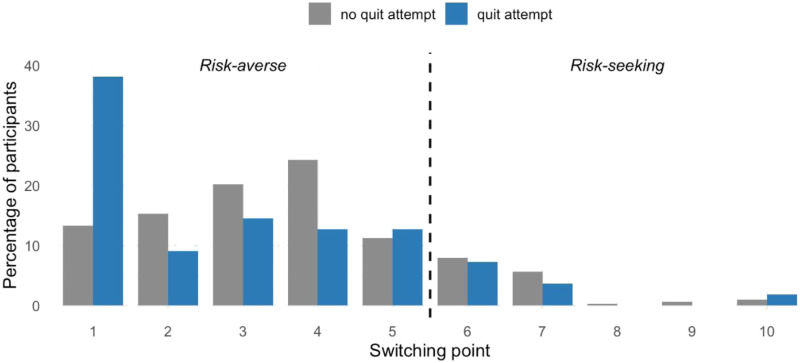
Distribution of risk preferences (switching points) by quit-attempt status. Data are presented as percentages. The dashed line at switching point 5.5 indicates the cut-off between risk-averse (≤5) and risk-seeking (>5) participants

The risk preference task revealed differences in the distribution of switching points between participants with and without a quit attempt (Fisher’s Exact Test, p-value = 0.011). Among those who had made a quit attempt, 38.2% chose the safe option already at the first decision (switching point = 1), indicating a risk-averse tendency, whereas non-attempters showed a more even distribution across higher switching points (Figure 1).

### 3.1. Regression Analysis

In the regression analyses, perceived dependence was the only factor significantly associated with quit attempts (Table 3). Participants who considered themselves unlikely to be dependent had higher odds of reporting a previous attempt compared with those who perceived dependence as likely (OR = 2.05, 95% CI: 1.26-3.32). No significant associations were observed for income, migration background, age, sex, education or concurrent use of other nicotine or tobacco products.

**Table 3.** Factors Associated With Quit Attempts

Variable	Attempts to stop smoking shisha yes vs. no (ref)
	OR (95% CI)
**Age** ^**‡**^	0.99 (0.96-1.03)
**Sex**	
Female (ref)	1
Male	1.07 (0.52-2.19)
**Educational qualification** ^**✜**^	
High (ref)	1
Middle	1.12(0.65-1.91)
Low	1.69(0.81-3.54)
**Income** ^**’**^	
High (>€2,000) (ref)	1
Middle (€1,001-2,000)	1.53 (0.78-2.99)
Low (<€1,001)	1.25 (0.67-2.33)
**Migration background** ^**Δ**^	
No (ref)	1
Yes	1.15 (0.71-1.88)
**Consumption quantity** ^**Ω**^	1.1 (1.00-1.02)
**Concurrent use** ^**∞**^	
Exclusive use (ref)	1
Concurrent use	0.88 (0.55-1.42)
**Perceived Dependence** ^**×**^	
Likely (ref)	1
Unlikely	2.05 (1.26-3.32)*
**Perceived Health Risks** ^***✜***^	
Harmful (ref)	1
Not Harmful	1.52 (0.66-3.47)
**Risk preferences** ^**#**^	
Risk averse (ref)	1
Risk seeking	0.79 (0.34-1.85)
**Time preferences 1 Month** ^*^	
Future-oriented (ref)	1
Variable discounting	0.97 (0.51-1.83)
Present-oriented	1.06 (0.39-2.84)
**Time preferences 6 Month** ^*^	
Future orientation (ref)	1
Variable discounting	0.66 (0.30-1.44)
Present-oriented	0.89 (0.42-1.89)

Data are presented as odds ratios (OR) with 95% confidence intervals (CI) around OR.*p < 0.05; **p < 0.01; ***p < 0.001.

**‡ Age** was included as a continuous variable.

^✜^**Educational qualification:** German equivalents to education qualification listed from lowest to highest: low=no qualification/junior high school equivalent, middle=secondary school equivalent, high=advanced technical college equivalent/high school equivalent.

**’Income:** Net monthly household income: low=<€1,000, middle=€1,001-2,000, and high=>€2,000.

^**Δ**^**Migration background:** Yes= one or both parents born abroad.

^**Ω**^**Consumption quantity:** Number of shisha heads used per month.

^**∞**^**Concurrent use:** Use of at least one additional product (cigarettes, e-cigarettes, or heated tobacco).

^**×**^**Perceived Dependence**: dichotomised into: Unlikely (includes “Very unlikely” and “Rather unlikely”), Likely (includes “Rather likely and “Very likely”).

^**✜**^**Perceived Health Risks:** dichotomised into: Harmful and Not harmful (includes “Yes, it harms my health a little”, “Yes, it harms my health somewhat” and “Yes, it harms my health a lot”).

^**#**^**Risk preferences:** classified as risk averse (switching point 1-5) or risk seeking (switching point 6-10).

*Time preferences (1- and 6-month task): future-oriented= always chose delayed reward, variable discounting=switched once, present-oriented=always chose delayed reward.

Perceived health risks were also not linked to quit attempts (OR = 1.52, 95% CI: 0.66-3.47). Finally, neither the distribution of risk averse and risk seeking individuals (OR = 0.79, 95% CI: 0.34-1.85) nor the distribution of present-, variable and future-oriented individuals at one or six months showed significant associations.

## 4. Discussion

In our sample, the majority of current shisha smokers had never tried to quit. Only about one in seven reported a previous quit attempt, and more than half did not consider themselves dependent. In addition, almost 40% used at least one additional tobacco or nicotine product.

The regression analysis showed that perceived dependence was the only factor significantly associated with quit attempts. Participants who did not perceive themselves as dependent were about twice as likely to have made a quit attempt compared with those who believed they were dependent. This aligns with evidence from other studies showing that lower levels of shisha dependence are linked to more frequent quit attempts and longer abstinence,^35,36^ while higher dependence is associated with less interest in quitting and shorter abstinence periods.^37^

This pattern may reflect different perceptions of autonomy and self-assessed dependence among shisha smokers. One contributing factor could be concurrent use of other tobacco or nicotine products, which is common in this group^17^ and has been shown to make quitting more difficult.^15,16^

Another possible explanation is that shisha smokers tend to underestimate their dependence, which in turn fosters a sense of control and confidence in their ability to quit. Previous studies have described a high confidence to quit among shisha smokers.^13^ This perception may be closely linked to low harm perception, as many shisha smokers view shisha as less addictive or less harmful than cigarettes.^13,18^ Taken together, this differs from patterns observed in cigarette smoking, where higher perceived dependence is often linked to a greater likelihood of quit attempts, suggesting that cessation behaviour may follow different patterns in shisha smokers. Consequently, individuals who perceive their use as low-risk or occasional may believe they could stop at any time, while those who recognise dependence may simultaneously feel less capable of quitting.

None of the other examined factors - sociodemographic characteristics, consumption-related factors or harm perception - were related to quit attempts. This contrasts with previous research about shisha smoking reporting associations between higher harm perception and quit intentions,^13,18^ but findings across the literature are mixed. One explanation may be differences in sample composition and data collection approaches, as previous studies vary considerably in terms of age groups, recruitment settings and patterns of shisha use.

Although behavioural economic variables were not statistically significant predictors, the descriptive results suggest potentially meaningful patterns. Participants who had tried to quit tended to prefer safer options, whereas those who had not tried were more evenly distributed across all choices. This aligns with theoretical expectations that individuals with higher risk aversion may be more responsive to perceived long-term health risks. The absence of significant associations might reflect limited statistical power within the subgroup of current shisha smokers or the complex nature of the underlying decision processes. Future research could explore whether risk and time preferences moderate the relationship between perceived dependence and quit motivation. Understanding how decision-making traits influence cessation behaviour may help tailor interventions that use reward framing or delayed gratification strategies to enhance motivation.

### 4.1. Strengths and Limitations

This is, to our knowledge, the first quantitative study in Germany focusing on shisha smoking and quit attempts. Participants were recruited through various online and offline channels, successfully reaching the target group of current shisha smokers. The integration of behavioural-economic measures such as risk and time preferences offers a novel approach to understanding cessation behaviour in this group.

However, some limitations should be considered. The study is based on a relatively small, non-representative sample, with a predominance of male participants. As all data were self-reported, recall or social desirability bias cannot be ruled out. Dependence was assessed using a single self-reported item rather than a validated instrument such as the Lebanon Waterpipe Dependence Scale, which limits the interpretation of dependence-related findings. As this study was designed as a pilot study, no a priori sample size calculation or power analysis was conducted. The online study design may introduce selective participation among individuals with regular internet access and those active on social media. In particular, recruiting a substantial proportion of participants via Instagram may have influenced the demographic and behavioural profile of the sample. The age distribution in the sample is consistent with previous research on shisha use but may limit the generalisability of the findings to older populations. Several variables were dichotomised for analysis, which may have reduced sensitivity. Although the SHISHA study follows a longitudinal design, the present analysis used cross-sectional baseline data and therefore cannot address causal or temporal relationships.

### 4.2. Conclusion

This study provides the first quantitative evidence on factors related to quit attempts among shisha smokers in Germany. Only a small proportion had ever tried to quit, and perceptions of dependence emerged as the main factor linked to cessation behaviour. The findings indicate that shisha smoking is frequently perceived as a low-risk, non-addictive activity, and that decisions to quit may follow patterns distinct from those observed in cigarette smoking.

From a public health perspective, these findings underline the importance of addressing misperceptions surrounding dependence and harm in shisha users. Targeted communication strategies should emphasise the addictive potential of shisha tobacco, even when used intermittently, and highlight its equivalence to cigarette smoking in terms of nicotine delivery and health risks. Interventions could also benefit from incorporating social and contextual aspects of shisha use - such as peer influence or the café setting - into cessation messaging.

Cessation strategies therefore need to move beyond traditional cigarette-focussed approaches. Tailored health education and prevention efforts should challenge misconceptions about dependence and harm while promoting practical, evidence-based cessation options that fit the social context of shisha use.

Further research is needed to better understand the motivational, cognitive, and contextual factors underlying shisha smoking and quitting. Future studies should combine representative and longitudinal data with qualitative methods to explore the psychological and social mechanisms in greater depth. Moreover, exploring behavioural-economic aspects such as risk and time preferences could provide new insight into why many users continue smoking despite low perceived dependence.

## Supplemental Material

Supplemental Material - Quitting Shisha Smoking Among Young Adults in Germany: Insights From an Online Survey on Sociodemographic, Perceptual and Behavioural Economic Factors Associated With Quit Attempts Supplemental Material for Quitting Shisha Smoking Among Young Adults in Germany: Insights From an Online Survey on Sociodemographic, Perceptual and Behavioural Economic Factors Associated With Quit Attempts by Cynthia Fedler, Daniel Kotz, Markus Vomhof, Stephanie Klosterhalfen, for the SHISHA-Study team in Tobacco Use Insights.

## Data Availability

This study used data from the SHISHA study. The dataset can be shared on reasonable request; researchers need to provide a short proposal describing how they intend to use it.*
