# Supplementary material for: Quitting Shisha Smoking Among Young Adults in Germany: Insights From an Online Survey on Sociodemographic, Perceptual and Behavioural Economic Factors Associated With Quit Attempts
Source: Tob Use Insights. 2026 Aug 7;19:1179173X261454173. doi: 10.1177/1179173X261454173 (PMC13451697; doi:10.1177/1179173X261454173)
Supplement: Supplementary file 1 [file sj-pdf-1-tui-10.1177_1179173X261454173.pdf]

Fragebogen - SHISHA-Studie – Aktuell Konsumierende Erwachsene

Anlage A – V10, 17.05.2024

Anmerkungen:

Die Fragen werden Erwachsenen im Alter ab 18 Jahren gestellt. Für Jugendliche im Alter von 14-17 wird eine weitere Version in „jugendgerechter“ Sprache angeboten („du“ statt „Sie“).

Um die Befragten nicht zu überfordern und die weitere Teilnahmebereitschaft zu erhöhen, soll das Ausfüllen des Online-Fragebogens maximal 20 Minuten in Anspruch nehmen. Sie finden nun alle Themenblöcke, die im gesamten Studienverlauf (Baseline + Follow-Up 1 + Follow-Up 2) behandelt werden sollen.

Die Fragen wurden für die unterschiedlichen Erhebungszeiträume mit den folgenden Farben markiert:

Baseline	Baseline und Follow Up 1 + 2	Follow Up 1	Follow Up 2

---

1) Wo sind Sie auf die Studie aufmerksam geworden?

1. Shisha-Bar
2. Im Rahmen einer anderen Studie, an der ich teilnehme
3. Soziale Medien
4. Schule/Jugendeinrichtung/Sportverein
5. Außenwerbung, wie z.B. Plakat/Litfaßsäule/Flyer
6. Freunde
7. Survey Circle
8. Sonstige (Freitext)

2) Alter: Wie alt sind Sie?

___________ Jahre

Falls 1)=3.: Über welche soziale Medien sind Sie auf unsere Studie aufmerksam geworden?

1. Instagram
2. TikTok
3. Facebook
4. Werbung bei Google
5. X

3) Filterfrage: Rauchen Sie Shisha (auch Wasserpfeife genannt)?

1. **Ja, bis heute**
2. Ja, regelmäßig, aber aktuell rauche ich keine mehr
3. Ja, früher mal, aber aktuell rauche ich keine mehr
4. Nein, ich habe noch nie eine Shisha geraucht
5. Ich möchte keine Angabe machen

Themenblock - Allgemeine Daten:

4) Welchem Geschlecht fühlen Sie sich zugehörig?

1. Männlich

2. Weiblich
3. Divers

59) Welchen höchsten Schulabschluss haben Sie oder streben Sie an?

1. Keinen allgemeinbildenden Schulabschluss
2. Hauptschulabschluss / Volksschulabschluss
3. Realschulabschluss / Mittlere Reife / Polytechnische Oberschule 10.Klasse
4. Fachhochschulreife
5. Abitur / Allgemeine Hochschulreife

6) Sind Sie in Deutschland geboren?

1. Ja
2. Nein
3. Ich möchte keine Angabe machen

7) Sind Ihr Vater UND Ihre Mutter in Deutschland geboren?

1. Ja, beide
2. Nein, nur ein Elternteil
3. Nein, kein Elternteil
4. Ich möchte keine Angabe machen

8) Wie viel Geld steht Ihnen monatlich zur freien Verfügung?

1. Weniger als 100€
2. 101 bis 500€
3. 501 bis 1000 €
4. 1.001 bis 2.000 €
5. 2.001 bis 3.000 €
6. 3.001 bis 4.000 €
7. Mehr als 4.000 €
8. Ich möchte keine Angabe machen

Themenblock - Rauchstatus/Konsumverhalten:

9) Wie alt waren Sie, als Sie das erste Mal Shisha geraucht haben?

1. Ca.__________ Jahre alt

10) Wie viele Köpfe rauchen Sie normalerweise in einem Monat?

1. Ca.__________ Köpfe pro Monat

11) Die Shisha kann mit tabakfreien Produkten (z.B. Dampfsteinen, Kräutermischungen, Gelen) oder auch mit Shishatabak befüllt werden. Zudem kann der Shishatabak aromatisiert sein (z.B. in den Geschmacksrichtungen fruchtig, süß, herb) oder nicht aromatisiert sein. Was rauchen Sie hauptsächlich?

1. Aromatisierten Shishatabak
2. Nicht aromatisierten Shishatabak
3. Tabakfreie Produkte
4. Ich weiß es nicht oder habe keine Vorliebe

12) Eine Shisha kann man in spezialisierten Bars (Shisha-Bars), aber auch an anderen Orten rauchen. Wo rauchen Sie diese? (Mehrfachantworten)

1. In speziellen Bars (z.B. Shisha-Bars/Shisha-Lounges)
2. Zu Hause oder bei Freunden (privat)
3. In der Öffentlichkeit (z.B. in Parks, an Seen etc.)
4. Im Ausland (z.B. Urlaubs- oder Geschäftsreise)
5. An sonstigen Orten

13) Wenn Sie sich Shishatabak besorgen, woher stammt dieser?

1. Von Freunden/Bekannten
2. Auf der Straße gekauft
3. Über Onlinehandel
4. Shisha-Shops
5. Tankstelle/Supermarkt/Kiosk
6. Sonstige Quelle ___________
7. Ich besorge mir selber keinen Shishatabak

14) Konsumieren Sie aktuell auch andere Tabak- und Nikotinprodukte oder nur Shisha? (Mehrfachantworten + Filterfrage)

1. Nur Shisha
2. Auch Zigaretten

   (a) Wie viele Zigaretten haben Sie ungefähr in den letzten 30 Tagen geraucht?

   Ungefähr __________ Zigaretten (Anzahl).

   (b) Wie alt waren Sie, als Sie das erste Mal Zigaretten geraucht haben?

   _________ Jahre

3. Auch E-Zigaretten

   (a) Falls Sie Einwegs- oder Pod-E-Zigaretten nutzen: Wie viele haben Sie in den letzten 30 Tagen genutzt?

   Ungefähr ________ Einweg- oder Pod-E-Zigaretten (Anzahl).

   (b) Falls Sie eine E-Zigarette mit Tank nutzen, wie viel Liquid (ml) haben Sie in den letzten 30 Tagen verbraucht?

   Ungefähr _________ml Liquid.

   (c) Wie alt waren Sie, als Sie E-Zigaretten das erste Mal genutzt haben?

   _________ Jahre

4. Auch Tabakerhitzer (z.B. IQOS)

   (a) Wie viele Tabak-Sticks haben Sie in den letzten 30 Tagen genutzt?

   Ungefähr __________Tabak-Sticks (Anzahl).

(b) Wie alt waren Sie, als Sie Tabakerhitzer das erste Mal verwendet haben?

_______Jahre

5. Auch Zigarillos und/oder Zigarren

(a) Wie viele Zigarillos/Zigarren haben Sie in etwa in den letzten 30 Tagen geraucht?

Ungefähr________ Zigarillos/ Zigarren (Anzahl).

(b) Wie alt waren Sie, als Sie Zigarillos/Zigarren das erste Mal geraucht haben?

_________Jahre

6. Auch Snus und/oder Nikotin-Pouches

(a) Wie viele Snus/Nikotin-Pouches haben Sie in den letzten 30 Tagen genutzt?

Ungefähr________Snus und/ oder Nikotin-Pouches (Anzahl)

(b) Wie alt waren Sie, als Sie Snus/Nikotin-Pouches das erste Mal verwendet haben?

________Jahre

15) Haben Sie schon einmal Cannabis mit der Shisha geraucht?

1. Ja
2. Nein
3. Ich möchte keine Angabe machen

16) Wie viel Geld geben Sie **pro Woche** schätzungsweise insgesamt für Tabak- und Nikotinprodukte aus? Gemeint sind alle Produkte, die Sie konsumieren, wie Zigaretten, E-Zigaretten, Shishas und so weiter, sowie deren Zubehör.

Freitext: ______________________

Abhängigkeit und gesundheitliche Einschränkungen:

Hinweis: Die folgenden beiden Fragen werden nur gestellt, wenn die Person angibt, ausschließlich Wasserpfeife zu konsumieren.

17) Wenn Sie jemand fragt: „Rauchen Sie?“, wie würden Sie die Frage beantworten?

1. Ja
2. Nein

18) Wenn Ihr Arzt/Ihre Ärztin Sie fragt: „Rauchen Sie?“, wie würden Sie die Frage beantworten?

1. Ja
2. Nein

19) Sind bei Ihnen jemals akute Probleme oder negative Wirkungen während oder nach dem Rauchen der Shisha aufgetreten?

1. Nein
2. Ja, akute gesundheitliche Probleme (wie z.B. Herzrasen, Übelkeit, Schwindel)
3. Ich weiß nicht

20) Wie wahrscheinlich ist es, dass Sie vom Shishatabak abhängig werden können /oder abhängig geworden sind?

1. Sehr unwahrscheinlich
2. Eher unwahrscheinlich
3. Eher wahrscheinlich
4. Sehr wahrscheinlich
5. Ich weiß nicht

21) Glauben Sie, dass es Ihrer Gesundheit schadet, wenn Sie Shisha rauchen?

1. Nein
2. Ja, es schadet meiner Gesundheit ein wenig
3. Ja, es schadet meiner Gesundheit etwas
4. Ja, es schadet meiner Gesundheit sehr
5. Ich weiß nicht

22) Bei einer Shisha besteht die Möglichkeit, diese allein oder aber mit weiteren Personen gemeinsam zu rauchen, z.B. durch das Herumreichen des gleichen Schlauchs oder das Anbringen weiterer Schläuche an derselben Shisha. Wie rauchen Sie Shisha hauptsächlich?

1. Ich rauche diese allein
2. Ich rauche diese mit weiteren Personen durch Nutzung <u>eines Schlauches</u>, der herumgereicht wird/wurde
3. Ich rauche diese mit weiteren Personen durch Nutzung <u>mehrerer Schläuche</u> an derselben Shisha
4. Ich rauche diese mit weiteren Personen durch Nutzung <u>mehrerer Schläuche</u>, die herumgereicht werden

23) Haben Sie schon einmal versucht, mit dem Rauchen der Shisha aufzuhören?

1. Ja, ich habe es bisher mindestens einmal versucht.
2. Nein, ich habe es bisher noch nicht versucht.
3. Nein, ich habe bisher noch nicht darüber nachgedacht
4. Ich möchte die Frage nicht beantworten.

24) Welche Faktoren würden Sie dazu motivieren, das Shisharauchen zu reduzieren oder aufzugeben? (Mehrfachantworten)

1. Wenn meine Freundinnen und Freunde nicht mehr Shisha rauchen
2. Wenn es nicht mehr zu mir passt
3. Wenn es mir zu teuer wird
4. Wenn es mir keine Freude mehr macht
5. Wenn ich dafür keine Zeit mehr habe
6. Gesundheitliche Probleme
7. Wenn ich merke, dass meine körperliche Fitness schlechter geworden ist
8. Wenn es gesetzlich verboten wäre
9. Wenn ich mehr Wissen über mögliche Gesundheitsschäden hätte
10. Wenn ich dafür eine Belohnung bekommen würde aufzuhören, z.B. Geld oder Gutscheine
11. Wenn meine Familie das nicht will
12. Wenn ich das Gefühl habe, abhängig zu sein
13. Sonstiges:_____________

25) Wie viel Shisha rauchen Sie im Vergleich zu anderen, die Shisha rauchen?

1. Ich rauche weniger als sie
2. Ich rauche mehr als sie
3. Ich rauche genauso viel wie sie

Regulierung durch den Preis:

26) Seit dem Jahr 2022 gibt es Steuererhöhungen für Shishatabak. Die Preise wurden teilweise bis zu 35% erhöht. Hat der Preisanstieg Ihr Rauchverhalten verändert?

1. Ja, ich habe weniger Shishatabak geraucht.
2. Nein, mein Rauchverhalten hat sich nicht verändert.
3. Ja, ich habe mehr Shishatabak geraucht.
4. Ich habe vor dem Jahr 2022 noch nicht geraucht.

Einfluss (Medien, soziales Umfeld):

27) Wenn Sie soziale Plattformen wie Instagram, TikTok, Facebook, X etc. nutzen, kommen Sie auch mit dem Thema „Shisha" in Berührung (z.B. Posts von Influencer:innen/Herstellern, Austausch mit anderen Shisharauchenden in Foren/Gruppen, etc.)?

1. Ja, Shishakonsum ist ständig Thema in den von mir genutzten sozialen Medien
2. Ja, Shisha ist teilweise Thema in den von mir genutzten sozialen Medien
3. Nein, Shishakonsum ist kein Thema in den von mir genutzten sozialen Medien
4. Ich nutze keine sozialen Medien

28) Konsumieren die Leute, mit denen Sie in Ihrer Freizeit gerne Zeit verbringen, aktuell ebenfalls nikotin- oder tabakhaltige Produkte, wie z.B. Zigaretten, E-Zigaretten, Shisha, Tabakerhitzer etc.?

1. Ja, und zwar folgende Produkte _________________________
2. Nein, keiner konsumiert aktuell nikotin- oder tabakhaltige Produkte
3. Ich weiß es nicht

29) Welche Musikrichtung hören Sie am liebsten?

1. (Deutsch) Rap/Hip Hop
2. Klassik
3. Jazz/Soul/Funk
4. Pop
5. Electro/Techno
6. Schlager
7. Metal/Rock/Punk
8. Sonstige _________________
9. Ich höre keine Musik

30) Warum rauchen Sie Shisha? Mehrfachantworten möglich.

1. Ich möchte zu einer Gemeinschaft gehören
2. Als alternatives Genussmittel statt Alkohol
3. Es schmeckt besser als Zigaretten
4. Ich glaube es ist weniger schädlich als Zigaretten
5. Meine Freunde rauchen es auch
6. In der Shisha-Bar ist es chillig
7. Ich kann mich dabei entspannen
8. Ich habe dann etwas zu tun / keine Langeweile mehr
9. Dies ist für mich eine soziale und kulturelle Tradition, die ich schätze
10. Sonstiges _______

Gesundheitskompetenzen:

31) Wie einfach oder schwierig ist es für Sie zu beurteilen, wie sich (Ihr) Shisharauchen auf (Ihre) Gesundheit auswirkt?

1. Sehr einfach
2. Einfach
3. Schwierig
4. Sehr schwierig

32) Haben Sie sich über die Auswirkungen der Shisha auf Ihre Gesundheit informiert? (Filterfrage)

1. Ja
2. Nein

33) Nur, wenn die Frage vorher mit ‚Ja' beantwortet wurde: Wie schwierig war es für Sie, Informationen über die Shisha im Zusammenhang mit Ihrer Gesundheit zu finden?

1. Sehr einfach
2. Eher einfach
3. Eher schwierig
4. Sehr schwierig

Wie möchtest du mehr über die Gesundheitsrisiken von Wasserpfeifen, Zigaretten oder E-Zigaretten erfahren? Du kannst mehrere Antworten auswählen.

1. Ich möchte gar keine Informationen dazu
2. Durch einen Arzt oder eine Ärztin
3. Durch Familienmitglieder
4. Durch meine Freunde oder Freundinnen
5. Durch Plakate in der Öffentlichkeit
6. Durch Krankenkassen
7. Durch meinen Sportverein
8. Durch Social Media Kanälen, wie zum Beispiel TikTok, Instagram, YouTube
9. Durch eine App oder Webseiten
10. Durch andere Personen/auf andere Weise

35) Einstellung zu Risiko

Wir möchten herausfinden, wie Ihre Einstellung zu Risiko ist. Um das herauszufinden, wählen Sie 10-mal eins von zwei Glücksrädern aus.

Welches Glücksrad würden Sie wählen? Es gibt keine richtigen oder falschen Antworten, aber leider auch kein Geld. ;-)

☐ **Option A**

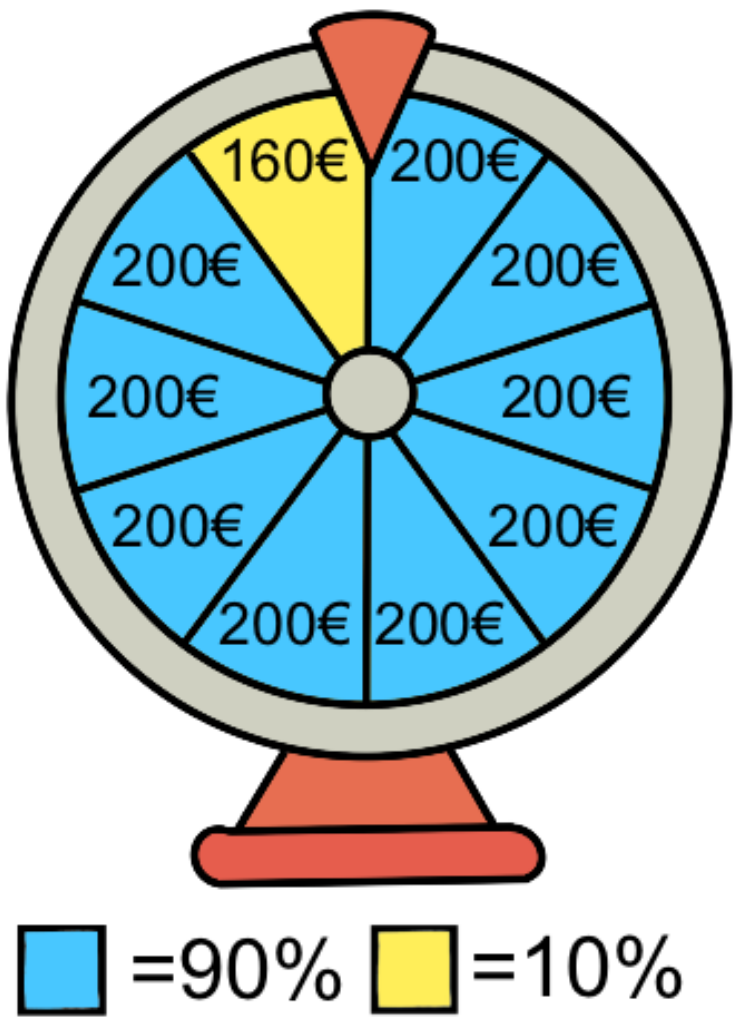

☐ **Option B**

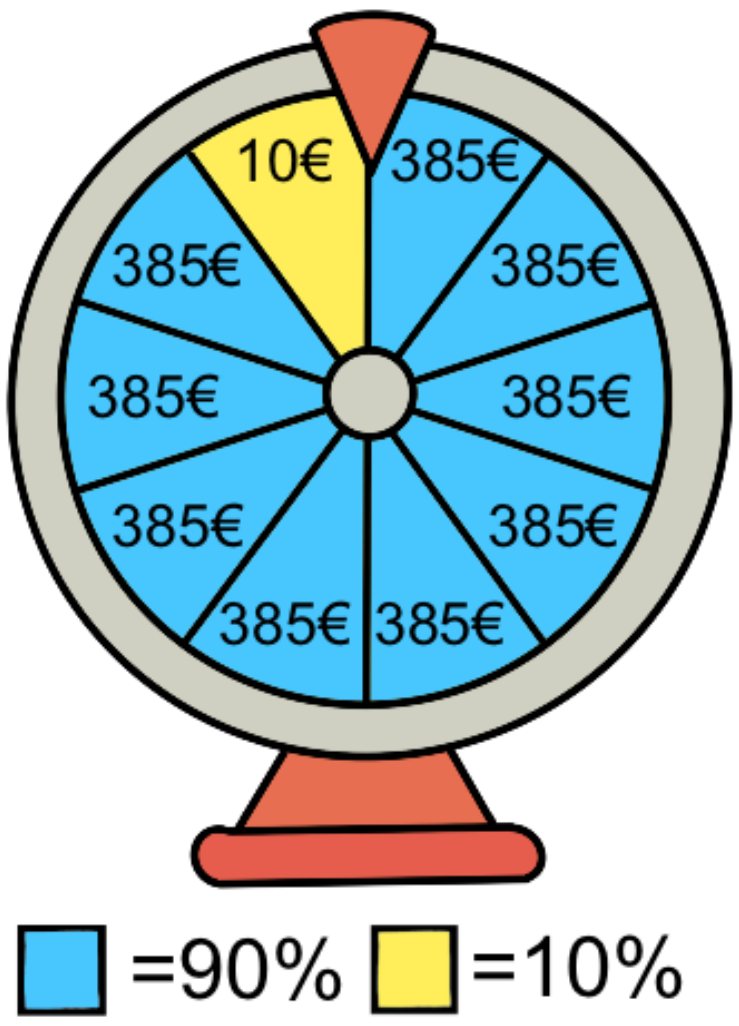

☐ **Option A**

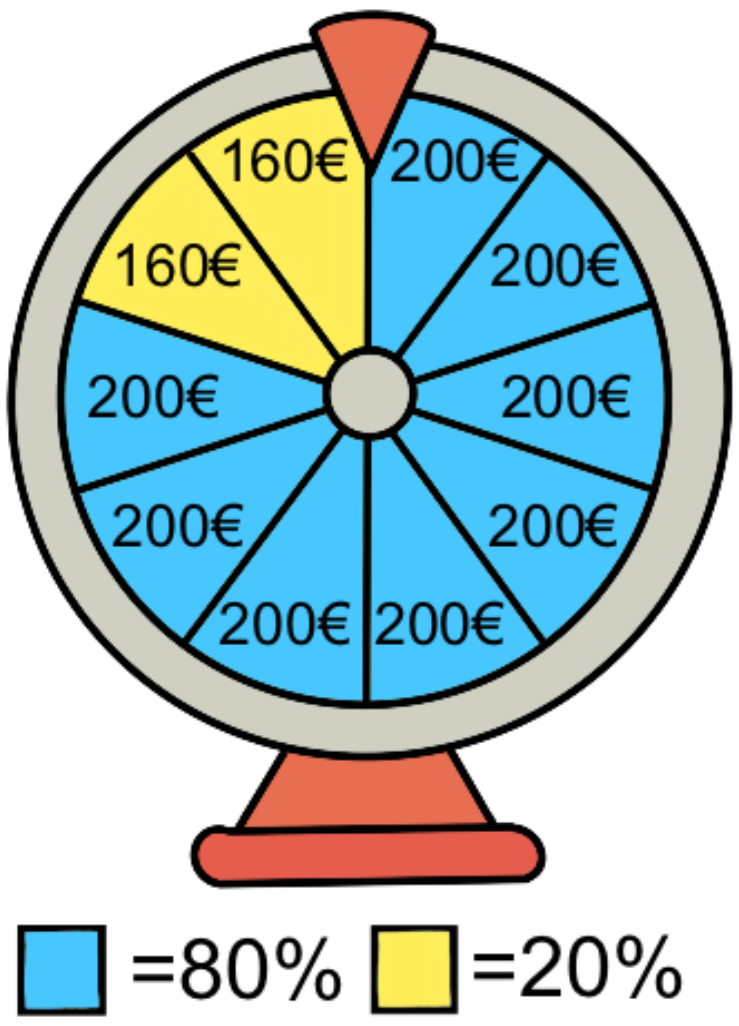

☐ **Option B**

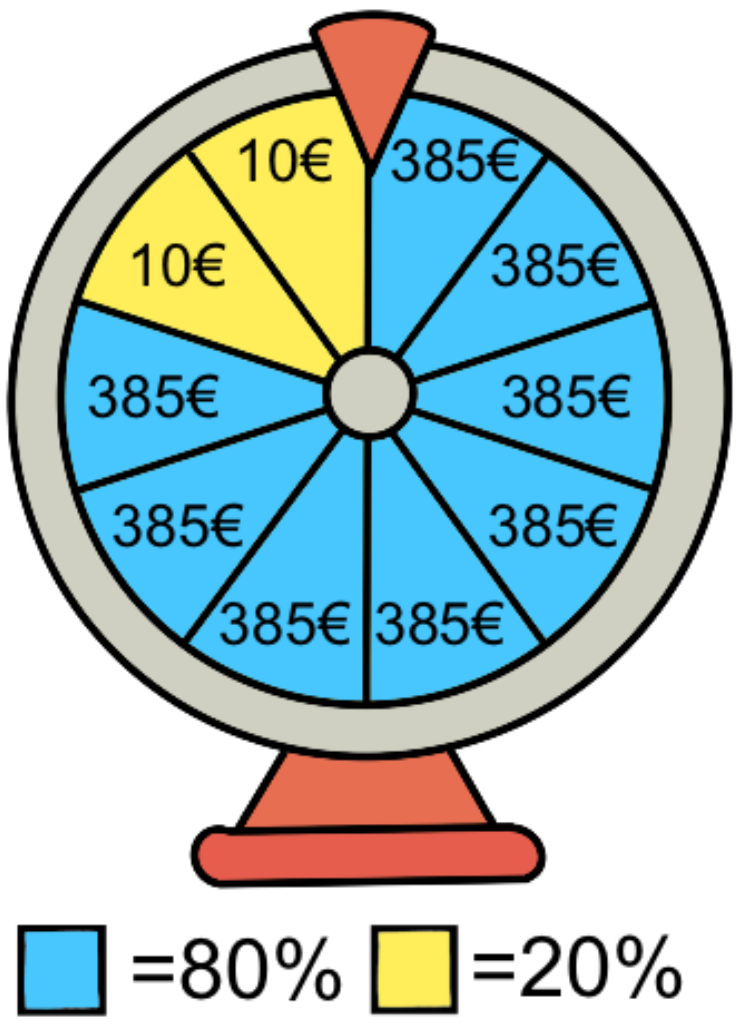

☐ **Option A**

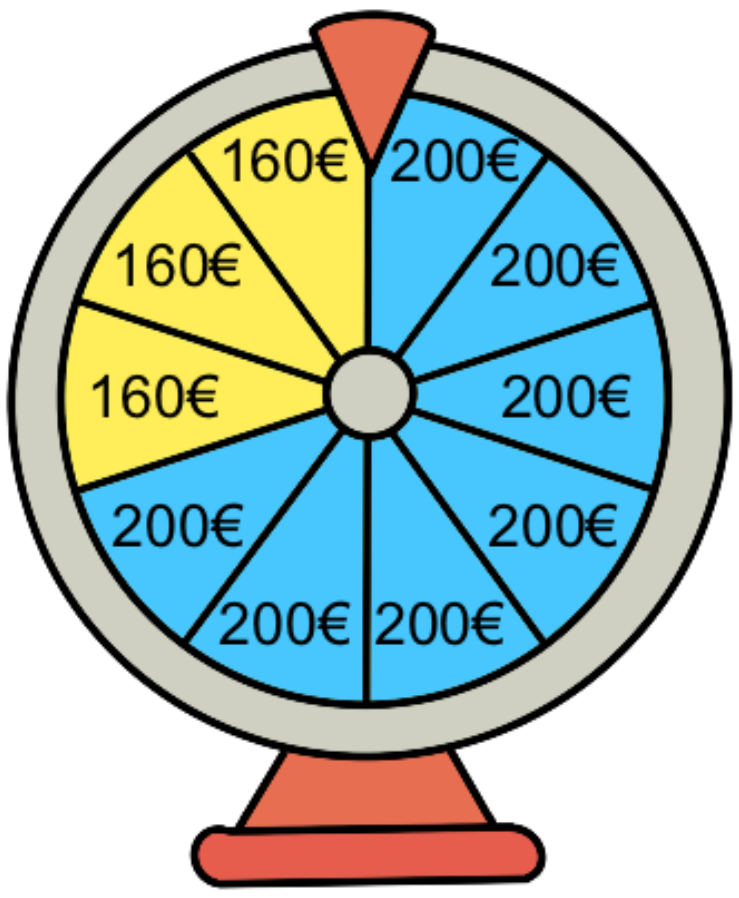

=70% =30%

☐ **Option B**

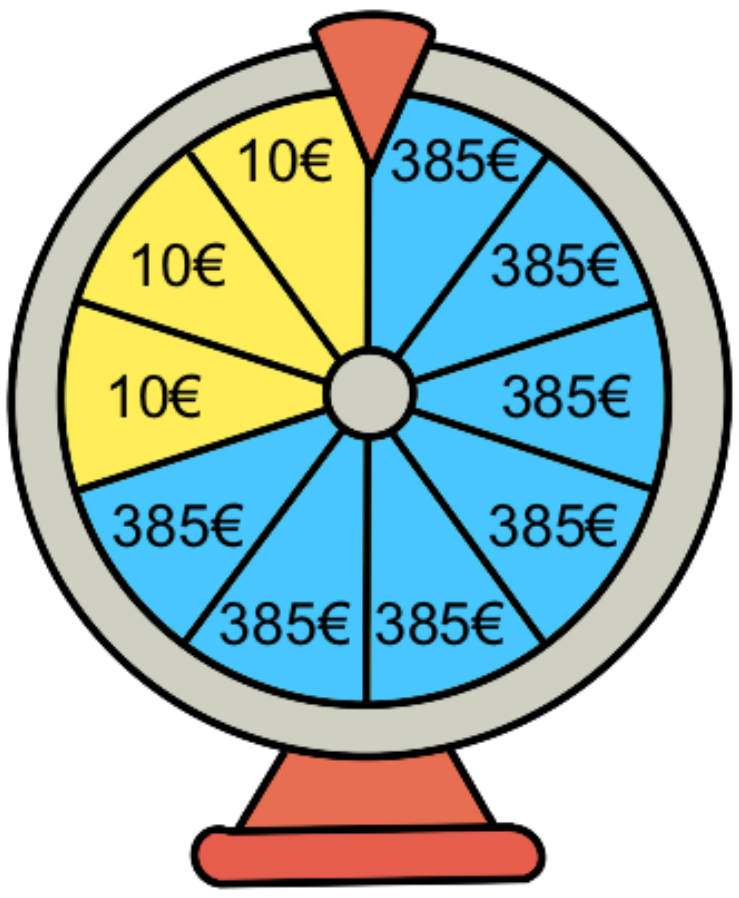

=70% =30%

☐ **Option A**

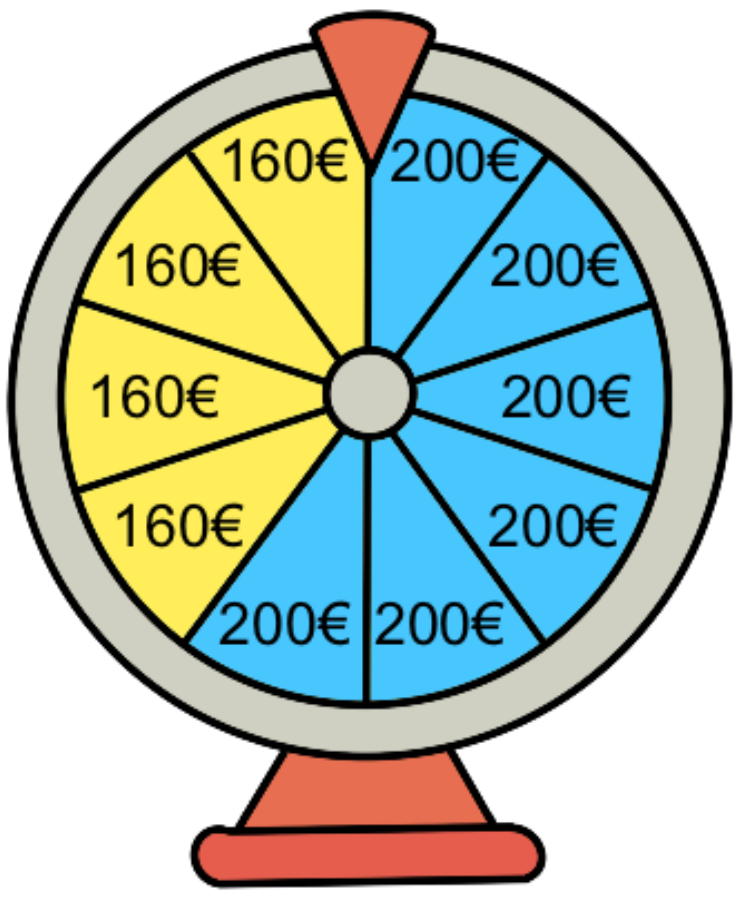

=60% =40%

☐ **Option B**

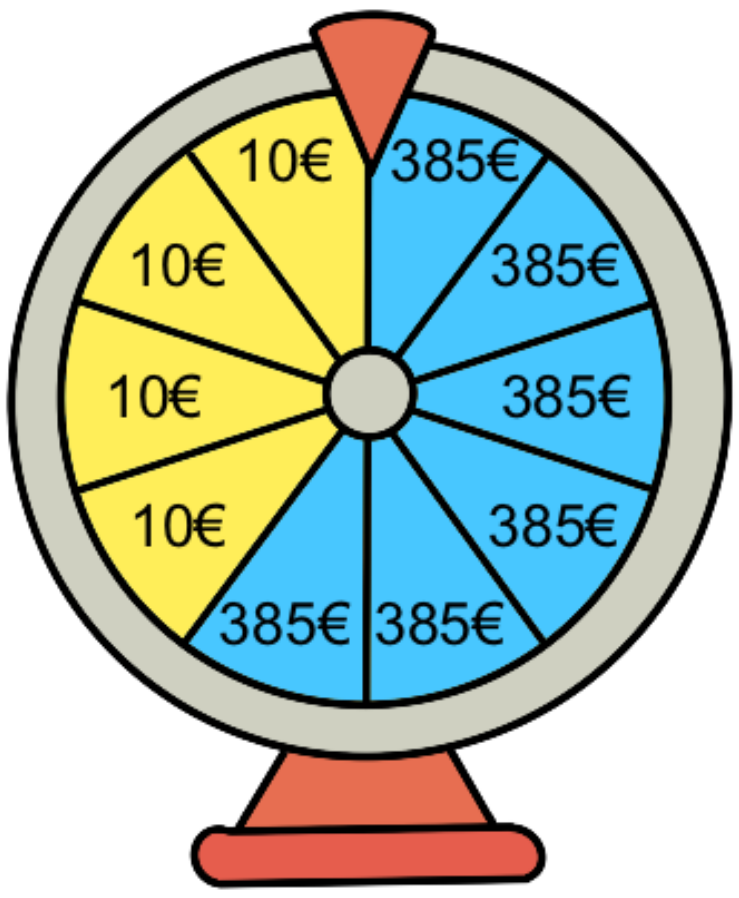

=60% =40%

☐ **Option A**

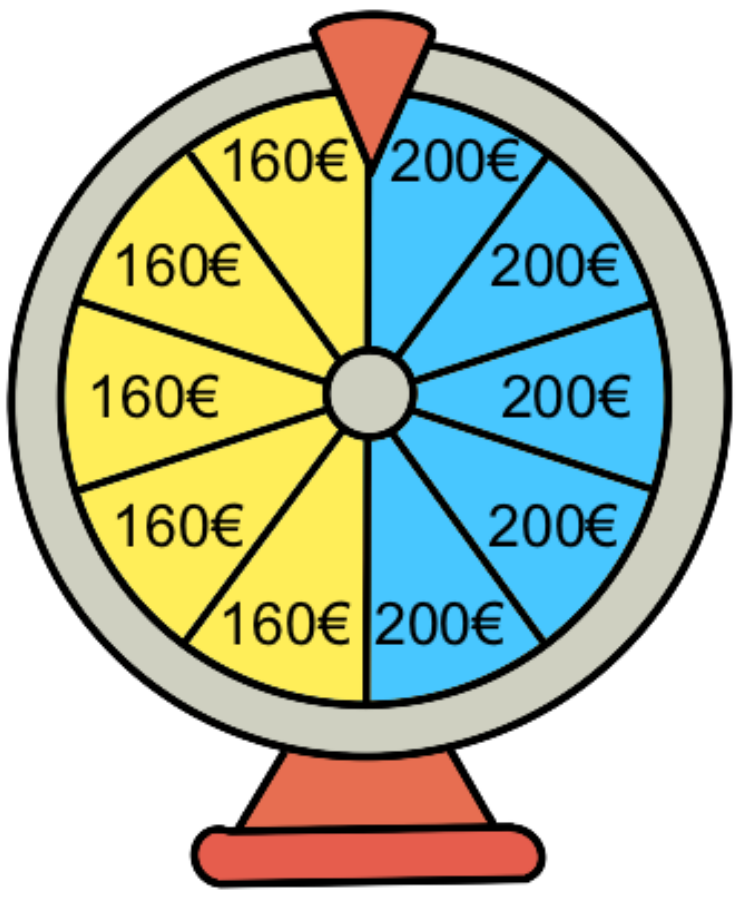

=50% =50%

☐ **Option B**

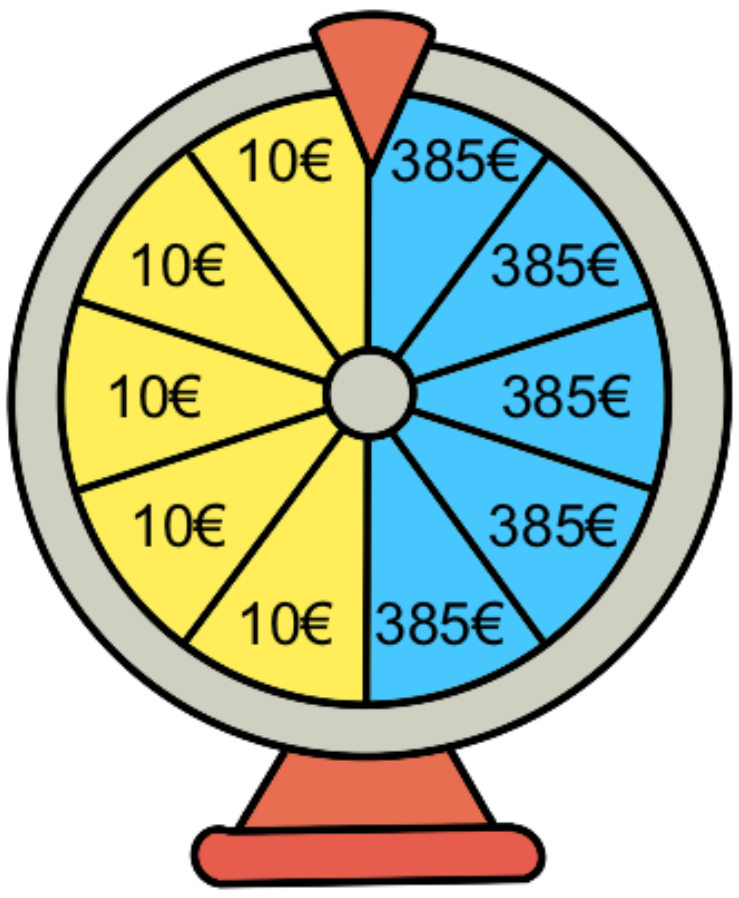

=50% =50%

☐ Option A	☐ Option B
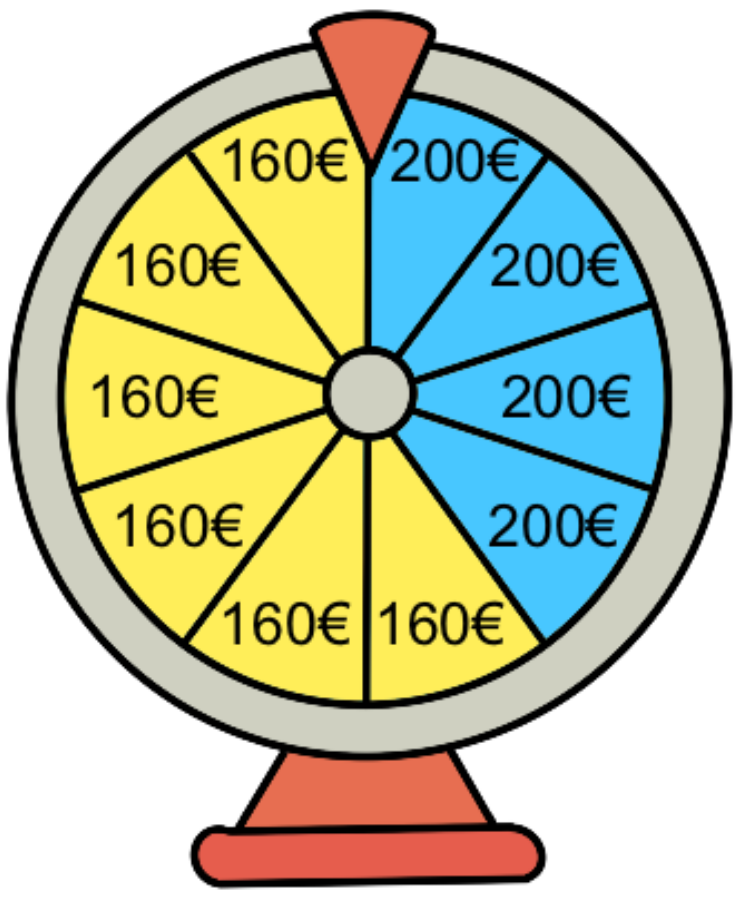<br>=40% =60%	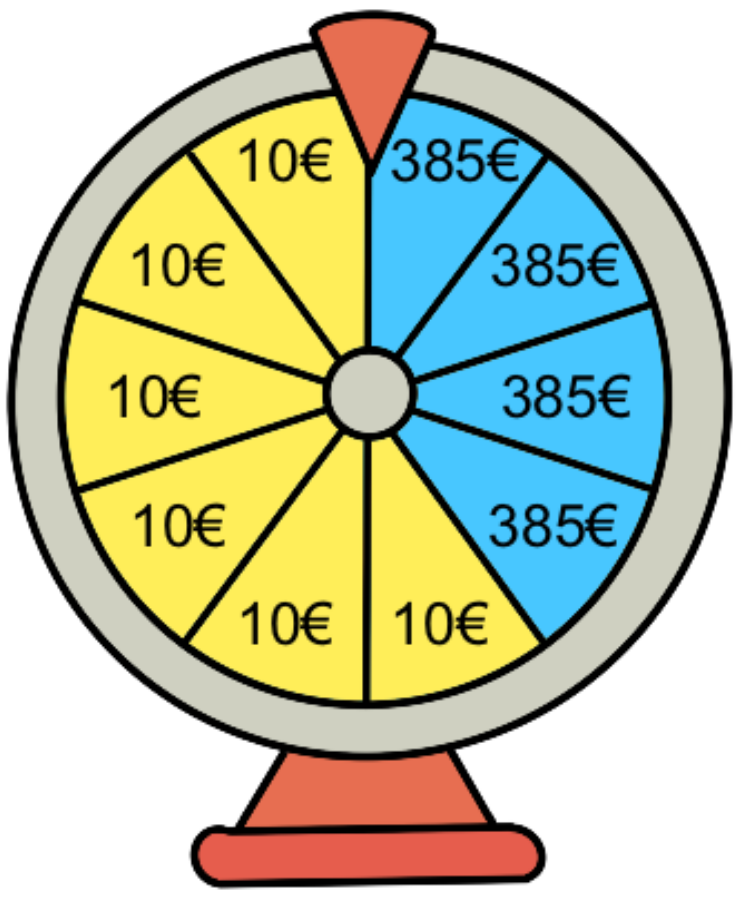<br>=40% =60%
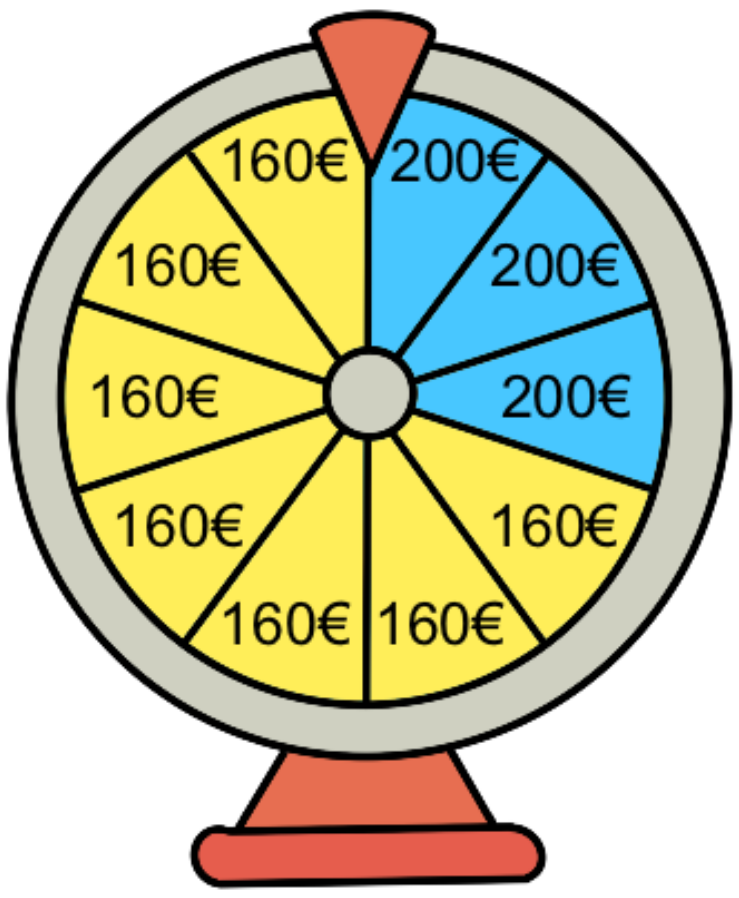<br>=30% =70%	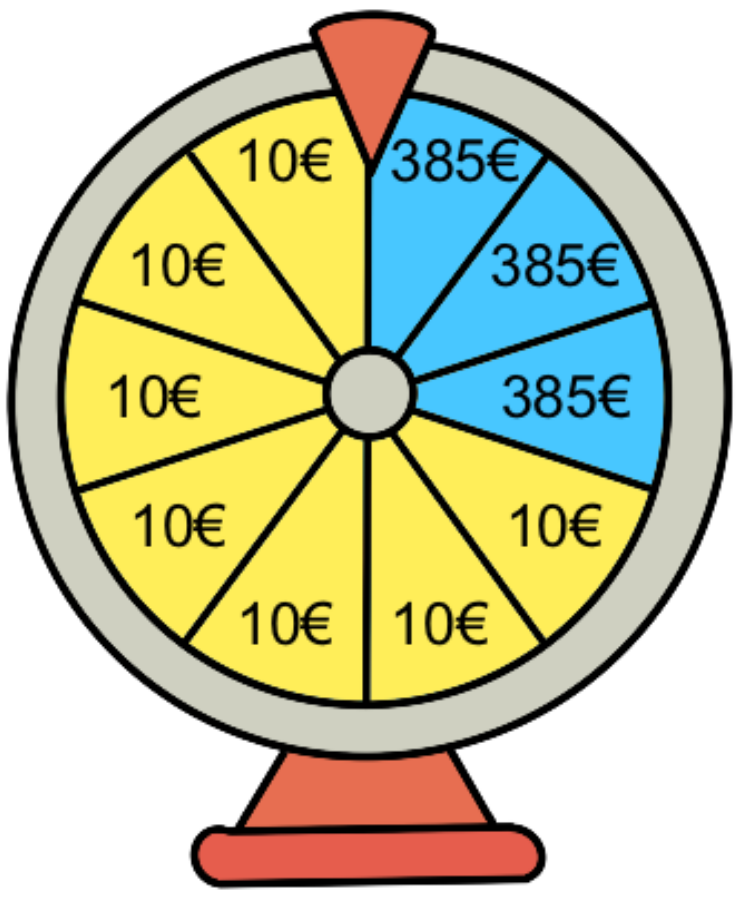<br>=30% =70%
☐ Option A	☐ Option B
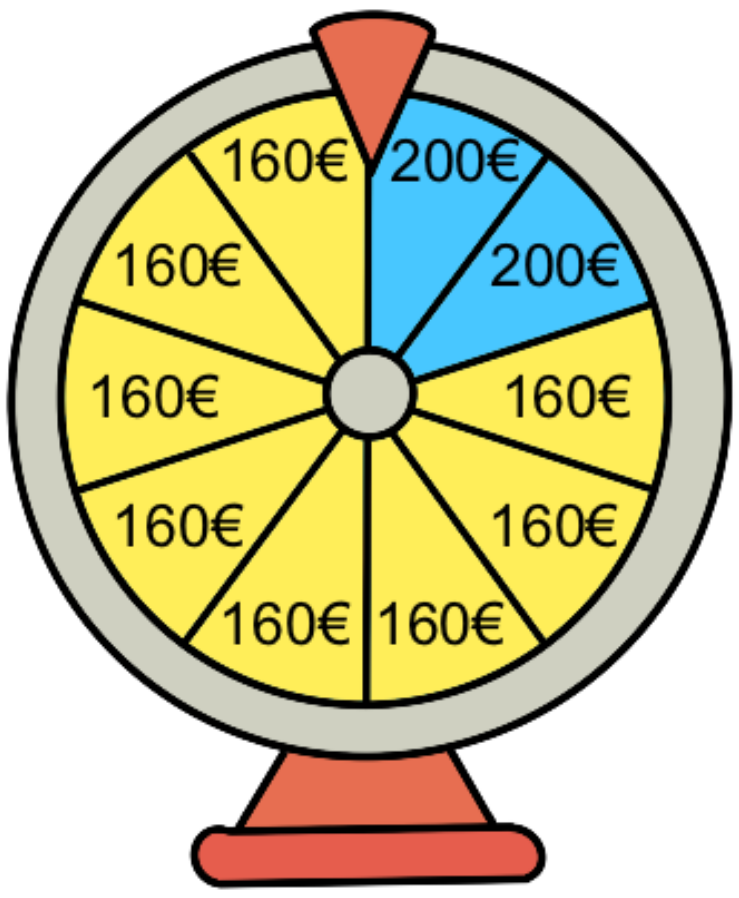<br>=20% =80%	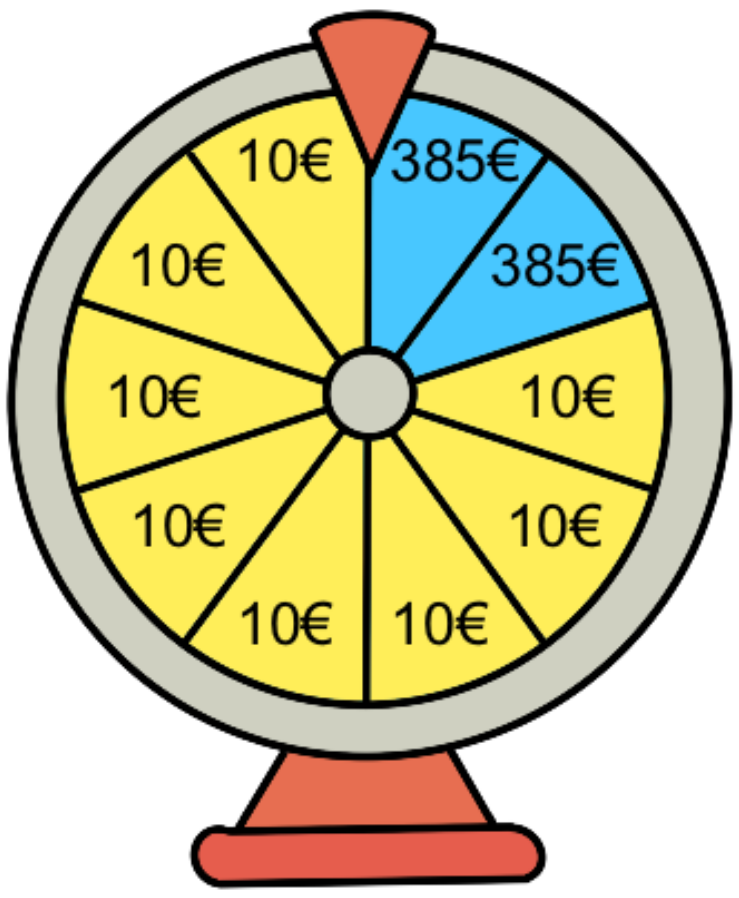<br>=20% =80%

☒ **Option A**

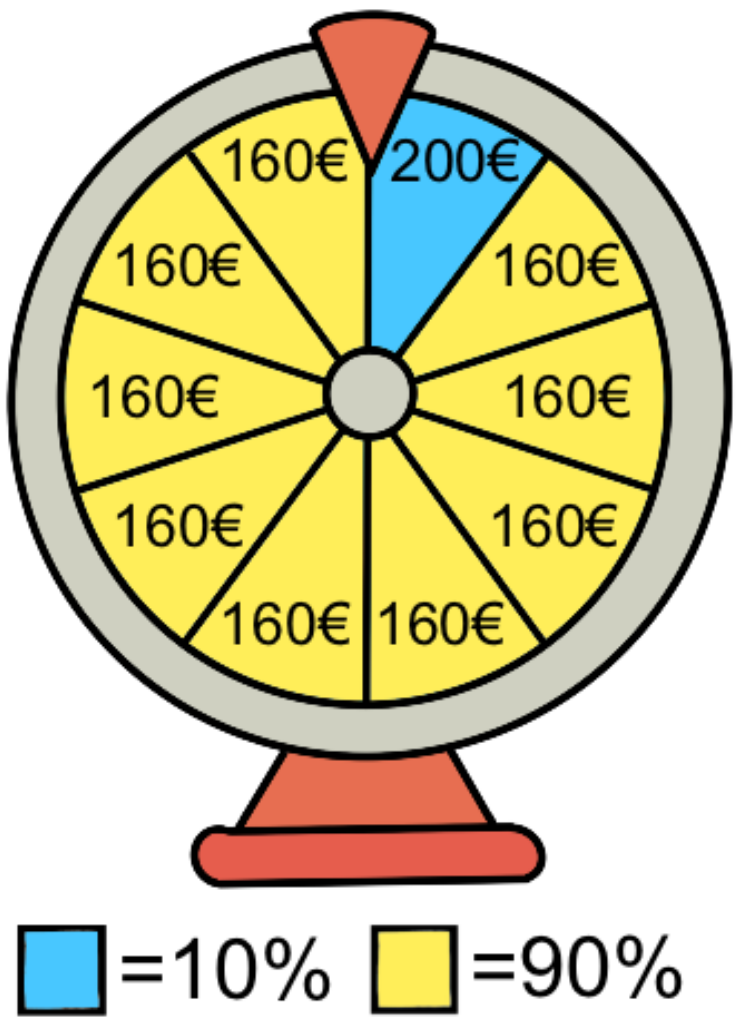

☐ **Option B**

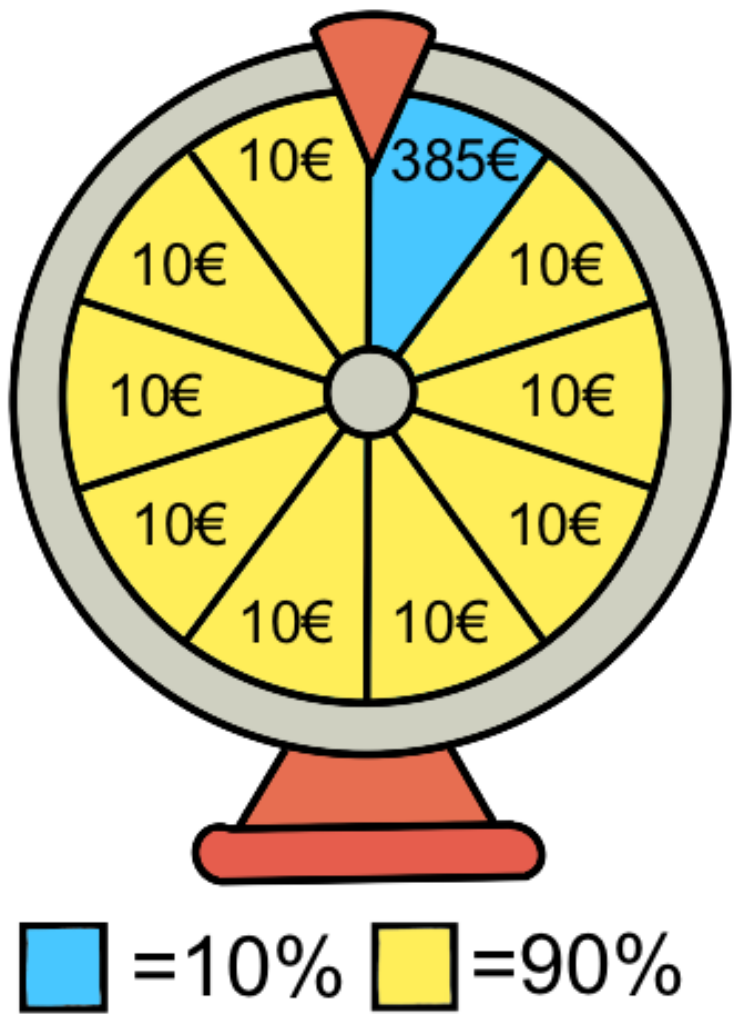

☐ **Option A**

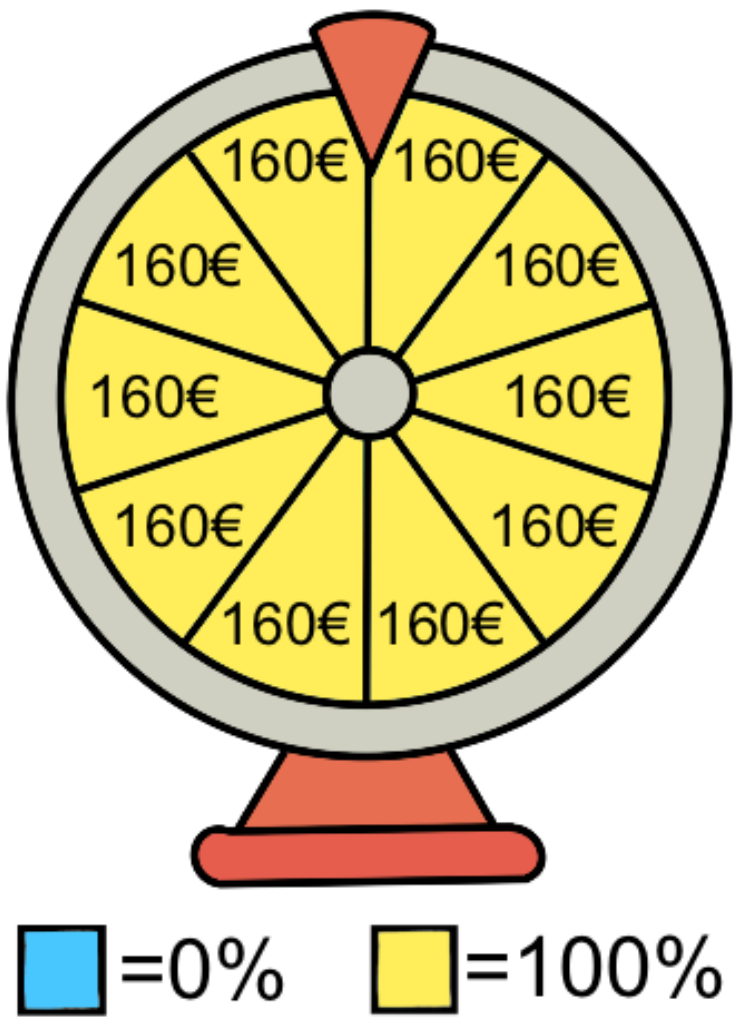

☐ **Option B**

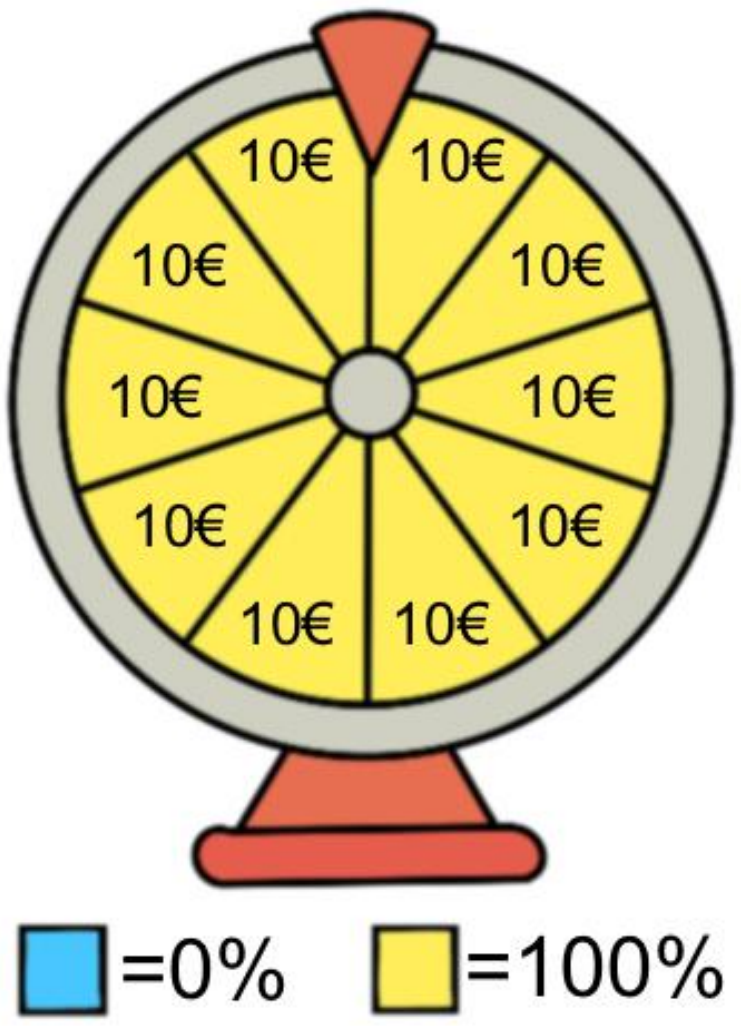

Welche der folgenden Aussagen sind wahr? Du kannst so viele Kästchen ankreuzen, wie du möchtest.

- ☐ Das Wasser in der Shisha filtert die Giftstoffe aus dem Rauch
- ☐ Shisha Rauchen kann süchtig machen
- ☐ Shishas sind weniger schädlich für die Gesundheit als Zigaretten
- ☐ Jede Art von Kohle ist für das Rauchen von Shishas geeignet
- ☐ Shishas können nur mit Shisha-Tabak verwendet werden
- ☐ Shisha-Tabak ist weniger schädlich als normaler Tabak von Zigaretten
- ☐ Shisha-Tabak enthält Vitamine
- ☐ Wenn man nur Shisha raucht und keine Zigaretten ist man trotzdem ein:e „Raucher:in“
- ☐ Personen unter 18 Jahren dürfen in einer Shisha-Bar eine Shisha rauchen
- ☐ Durch das Teilen eines gemeinsamen Mundstücks beim Shisha-Rauchen kann man krank werden

Zeitpräferenzen

In der nachfolgenden Tabelle sehen Sie jeweils zwei Optionen (Option A und Option B). Stellen Sie sich vor, dass Sie sich immer für eine dieser Optionen entscheiden müssen. Geben Sie für jede der 16 Entscheidungen an, welche Option Ihnen besser gefällt.

Nr.	Option A		Option B
1)	Sie erhalten **300 €** sofort	☐ ☐	Sie erhalten **307 €** in einem Monat
2)	Sie erhalten **300 €** sofort	☐ ☐	Sie erhalten **314 €** in einem Monat
3)	Sie erhalten **300 €** sofort	☐ ☐	Sie erhalten **321 €** in einem Monat
4)	Sie erhalten **300 €** sofort	☐ ☐	Sie erhalten **328 €** in einem Monat
5)	Sie erhalten **300 €** sofort	☐ ☐	Sie erhalten **335 €** in einem Monat
6)	Sie erhalten **300 €** sofort	☐ ☐	Sie erhalten **342 €** in einem Monat
7)	Sie erhalten **300 €** sofort	☐ ☐	Sie erhalten **349 €** in einem Monat
8)	Sie erhalten **300 €** sofort	☐ ☐	Sie erhalten **356 €** in einem Monat

Entscheiden Sie sich bitte auch hier zwischen jeweils zwei Optionen. Diesmal beträgt die Wartezeit nicht einen Monat, sondern **sechs Monate.**

9)	Sie erhalten **300 €** sofort	☐ ☐	Sie erhalten **307 €** in 6 Monaten
10)	Sie erhalten **300 €** sofort	☐ ☐	Sie erhalten **314 €** in 6 Monaten
11)	Sie erhalten **300 €** sofort	☐ ☐	Sie erhalten **321 €** in 6 Monaten
12)	Sie erhalten **300 €** sofort	☐ ☐	Sie erhalten **328 €** in 6 Monaten
13)	Sie erhalten **300 €** sofort	☐ ☐	Sie erhalten **335 €** in 6 Monaten
14)	Sie erhalten **300 €** sofort	☐ ☐	Sie erhalten **342 €** in 6 Monaten
15)	Sie erhalten **300 €** sofort	☐ ☐	Sie erhalten **349 €** in 6 Monaten
16)	Sie erhalten **300 €** sofort	☐ ☐	Sie erhalten **356 €** in 6 Monaten

Geschafft!! Vielen Dank für Ihre Teilnahme. Wir würden Sie bitten, einen ähnlichen Fragebogen noch **einmal in 2 Monaten** und noch **einmal in 4 Monaten** online auszufüllen. Falls Sie damit einverstanden sind, geben Sie einfach hier Ihre E-Mail-Adresse ein, damit wir Sie kontaktieren können. Als Dankeschön dafür, dass Sie uns Ihre Zeit schenken, erhalten Sie nach dem Ausfüllen des Fragebogens in 2 und 4 Monaten die Möglichkeit, **Gutscheine im Wert von bis zu 50€ zu gewinnen**.

E-Mail-Adresse: ____

Unser Ziel ist es, gemeinsam mit Ihnen mehr über den Shishakonsum in Deutschland zu erfahren. Falls Sie an den 2 Folgebefragungen **nicht weiter teilnehmen möchten**, seien Sie so nett und geben uns Ihren Hauptgrund dafür an:

1. Der Fragebogen war zu lang / es hat zu lange gedauert
2. Die Fragen waren nicht verständlich
3. Die Fragen waren thematisch nicht interessant für mich
4. Die technische Umsetzung gefällt mir nicht
5. Sonstiges: ______________________
6. Ich möchte die Frage nicht beantworten.

Wenn Sie Kommentare, Anmerkungen oder ähnliches zu diesem Fragebogen haben, können Sie dies hier gerne mitteilen. Gibt es beispielsweise zu Ihrem Shishakonsum etwas, was Ihnen wichtig ist und was in diesem Fragebogen jedoch noch nicht erwähnt wurde, dann teilen Sie uns dies bitte mit. – Vielen Dank
